# Draft Genome Sequences for 10 Isolates of the Swine Pathogen *Haemophilus parasuis*

**DOI:** 10.1128/genomeA.00739-13

**Published:** 2013-09-19

**Authors:** Joanna S. Kuehn, Karen B. Register, Gregory J. Phillips

**Affiliations:** Department of Veterinary Microbiology and Preventive Medicine, College of Veterinary Medicine, Iowa State University, Ames, Iowa, USAa; Ruminant Diseases and Immunology Research Unit, Agricultural Research Service, National Animal Disease Center, Ames, Iowa, USAb

## Abstract

*Haemophilus parasuis* colonizes the upper respiratory tract of swine and can cause a severe systemic disease known as Glässer’s disease. We report here the draft genome sequences of 10 isolates from geographically diverse locations representing the full virulence spectrum of the microorganism, which will aid in understanding the pathobiology of *H. parasuis*.

## GENOME ANNOUNCEMENT

*Haemophilus parasuis* is a Gram-negative microorganism that colonizes the upper respiratory tract of pigs, where it can cause a severe systemic infection known as Glässer’s disease. This disease is characterized by fibrinous polyserositis (inflammation of the serous membranes), arthritis, and meningitis. At least 15 serovars of *H. parasuis* have been recognized, and untypeable strains are also common (1). *H. parasuis* is an important pathogen in all major swine-raising countries throughout the world, especially in age-segregated production systems, where it is responsible for significant economical losses due to mortality and lost production.

Until relatively recently, no genomic sequence data for *H. parasuis* have been available. The initial release of the draft sequence of *H. parasuis* 29755 in GenBank (accession no. ABKM00000000), done by Roche/454 pyrosequencing (2), was followed by the complete genome sequence of *H. parasuis* SH0165 (3); these sequences have been valuable resources to researchers of this pathogen (4–6). However, these initial genome sequences provide a limited reference, as they both represent virulent serovar 5 strains. The additional draft sequences of the strains presented here, including a second genomic sequence of strain 29755, include multiple serovars and virulence phenotypes that will likely lead to the discovery of virulence mechanisms, as well as improved vaccines for disease prevention.

Based on sequences obtained using Illumina GAIIx whole-genome shotgun sequencing, we announce the availability of draft sequences for the following 10 *H. parasuis* strains and their serovars: 29755 (serovar 5), Nagasaki (serovar 5), SW114 (serovar 3), MN-H (serovar 13), 12939 (serovar unknown), 84-15995 (serovar 15), H465 (serovar 11), D74 (serovar 9), 174 (serovar 7), and SW140 (serovar 2). Strains Nagasaki, SW114, 84-15995, H465, D74, 174, and SW140 are serovar reference strains (1). Sequencing libraries were prepared from genomic DNA using an Illumina paired-end DNA sample preparation kit according to the manufacturer’s instructions, with a modification to the gel extraction incubation temperature (7). Each library was run on a single sequencing lane for 2 × 75 (strains SW114, 12939, 84-15995, H465, and D74) or 2 × 100 (strains Nagasaki, MN-H, 29755, 174, and SW140) cycles. *De novo* paired-end assembly was performed using the NextGENe assembler version 2.00 (Softgenetics). A total of 15,227,504 to 36,219,053 reads per strain were assembled and produced sequences for which the estimated coverage exceeded 100-fold for each strain. NextGENe ContigMerge was used to assemble contigs. The average G+C percentages of the drafts range from 39.6% to 39.9%. The genome sequences were annotated using the Institute for Genome Sciences (IGS) Annotation Engine (8). The features of the *H. parasuis* draft genomes are summarized in Table 1. Additional characterization of these *H. parasuis* strains is forthcoming.

**TABLE 1:** Genome features and accession no. of the sequenced strains

Strain	Accession no.	No. of contigs	Avg. contig length (bp)	N_50_ (bp)	Genome size (Mb)	No. of proteins
29755	ABKM00000000	96	15,760	52,793	1.51	2,127
Nagasaki	APBT00000000	78	27,930	69,670	2.18	2,308
SW114	APBU00000000	98	19,781	83,504	1.94	1,947
MN-H	APBV00000000	82	21,476	68,488	1.76	1,859
12939	APBW00000000	76	25,697	48,588	1.95	1,974
84-15995	APBX00000000	73	28,641	59,326	2.09	2,206
H465	APBY00000000	94	15,941	46,667	1.50	1,540
D74	APBZ00000000	68	32,787	147,970	2.23	2,218
174	APCA00000000	63	21,743	48,645	1.37	1,433
SW140	APCB00000000	77	18,356	84,269	1.41	1,484

### Nucleotide sequence accession numbers.

This whole-genome shotgun project has been deposited at DDBJ/EMBL/GenBank under the accession no. shown in Table 1.

## References

[1] Kielstein P Rapp-Gabrielson VJ 1992 Designation of 15 serovars of *Haemophilus parasuis* on the basis of immunodiffusion using heat-stable antigen extracts. J. Clin. Microbiol. 30:862–865 1572971 10.1128/jcm.30.4.862-865.1992 PMC265175

[2] Mullins MA Register KB Bayles DO Dyer DW Kuehn JS Phillips GJ 2011 Genome sequence of *Haemophilus parasuis* strain 29755. Stand. Genomic. Sci. 5:61–68 22180811 10.4056/sigs.2245029 PMC3236040

[3] Yue M Yang F Yang J Bei W Cai X Chen L Dong J Zhou R Jin M Jin Q Chen H 2009 Complete genome sequence of *Haemophilus parasuis* SH0165. J. Bacteriol. 191:1359–1360 19074396 10.1128/JB.01682-08 PMC2632009

[4] Xu Z Yue M Zhou R Jin Q Fan Y Bei W Chen H 2011 Genomic characterization of *Haemophilus parasuis* SH0165, a highly virulent strain of serovar 5 prevalent in China. PLoS One 6:e19631. 10.1371/journal.pone.0019631 21611187 PMC3096633

[5] Mullins MA Register KB Bayles DO Loving CL Nicholson TL Brockmeier SL Dyer DW Phillips GJ 2009 Characterization and comparative analysis of the genes encoding *Haemophilus parasuis* outer membrane proteins P2 and P5. J. Bacteriol. 191:5988–6002 19633080 10.1128/JB.00469-09 PMC2747881

[6] Howell KJ Weinert LA Luan S-L Peters SE Chaudhuri RR Harris D Angen Ø Aragon V Parkhill J Langford PR Rycroft AN Wren BW Tucker AW Maskell DJ on behalf of the BRADP1T Consortium 2013 Gene content and diversity of the loci encoding biosynthesis of capsular polysaccharides of the 15 serovar reference strains of *Haemophilus parasuis*. J. Bacteriol. 195:4264–4273 23873912 10.1128/JB.00471-13 PMC3754760

[7] Quail MA Kozarewa I Smith F Scally A Stephens PJ Durbin R Swerdlow H Turner DJ 2008 A large genome center’s improvements to the Illumina sequencing system. Nat. Methods 5:1005–1010 19034268 10.1038/nmeth.1270 PMC2610436

[8] Galens K Orvis J Daugherty S Creasy HH Angiuoli S White O Wortman J Mahurkar A Giglio MG 2011 The IGS standard operating procedure for automated prokaryotic annotation. Stand. Genomic. Sci. 4:244–251 21677861 10.4056/sigs.1223234 PMC3111993

